# EUCAST olorofim MICs for 3,550 Danish mold and dermatophyte isolates from 2020 to 2023

**DOI:** 10.1128/aac.00353-25

**Published:** 2025-06-26

**Authors:** Joseph Meletiadis, Karin Meinike Jørgensen, Karen Marie Thyssen Astvad, Nissrine Abou-Chakra, Maiken Cavling Arendrup

**Affiliations:** 1 Unit of Mycology, Statens Serum Institut 4326, Copenhagen, Denmark; 2 Clinical Microbiology Laboratory, Attikon University Hospital, National and Kapodistrian University of Athens 68993, Athens, Greece; 3 Department of Clinical Microbiology, Rigshospitalet 53146, Copenhagen, Denmark; 4 Department of Clinical Medicine, University of Copenhagen 652984, Copenhagen, Denmark; University Children's Hospital Münster, Münster, Germany

**Keywords:** EUCAST AFST, olorofim, mold, dermatophyte, antifungal susceptibility testing, antifungal agents

## Abstract

The dihydroorotate dehydrogenase (DHODH) inhibitor olorofim has potent activity against most molds. Recent reports of *in vitro* acquired resistance to olorofim and an agrochemical fungicide DHODH inhibitor warrant continuous monitoring of *in vitro* activity of olorofim. We examined the activity of olorofim against a contemporary, large collection of molds and explored the impact of different dilution schemes used for the preparation of microdilution plates. In total, 3,550 isolates referred to Statens Serum Institut during 2020–2023 were analyzed. Isolates were identified with matrix-assisted laser desorption/ionization time-of-flight mass spectrometry (MALDI-TOF MS) and/or sequencing. MICs of olorofim, amphotericin B, itraconazole, posaconazole, voriconazole, isavuconazole, and terbinafine were determined using EUCAST E.Def 9.4 and E.Def 11.0 protocols. Drug dilutions were prepared either using serial dilutions directly in the medium (Serial, 2020–2023) or according to the ISO standard 20776-1, with predilutions in DMSO before dilution in medium (introduced during 2023). For isolates with elevated MICs to azoles and olorofim, *Cyp51A*, *Hmg1,* and *PyrE* were sequenced as deemed relevant. Serial dilutions generated MICs 1–2 dilutions higher than the ISO dilutions for olorofim, voriconazole, and isavuconazole. Olorofim MICs were <0.5 mg/L (modal MICs 0.03–0.5 mg/L) against most isolates of *Aspergillus*, *Fusarium*, *Scedosporium*, *Talaromyces*, *Penicillium*, *Paecilomyces*, and dermatophytes. Exceptions included isolates belonging to *Aspergillus section Aspergillus,* to *Fusarium dimerum* and *Fusarium solani* species complexes, and some rare molds (MICs > 1 mg/L). Olorofim susceptibility was stable over the years and not affected by Cyp51A, Hmg1, or PyrE Q35L alterations. Olorofim maintained broad, potent *in vitro* activity over the years against a contemporary collection of molds.

## INTRODUCTION

Olorofim is an orotomide compound currently being tested in a Phase 3 clinical trial (ClinicalTrials.gov Identifier: NCT05101187). It has good *in vitro* activity against various molds as previously demonstrated (1). Its mechanism of action is unique as it targets the fungal class 2 dihydroorotate dehydrogenase (DHODH) enzyme involved in *de novo* pyrimidine biosynthesis (2). It has activity against different mold species, including *Scedosporium* and some *Fusarium* spp.*,* which are difficult to treat with current agents (3), but it has no activity against yeasts and Mucorales due to differences in their DHODH enzymes compared to susceptible fungi (2, 4). We have recently reported potent activity against *Aspergillus fumigatus*, including azole-resistant isolates, and other *Aspergillus* species, such as *Aspergillus terreus*, *Aspergillus nidulans*, *Aspergillus niger*, *Aspergillus flavus* species complex (SC), and the difficult-to-treat *Aspergillus calidoustus* and *Aspergillus tubingensis* (5). Moreover, we found that comparable olorofim MICs were obtained independently of whether drug dilutions were prepared using serial dilution in medium in the plate or the ISO standard 20776-1-based predilution in DMSO in tubes, dilution in medium in tubes, followed by transfer to the plate (6). Although resistant isolates have not been found so far among clinical isolates, recent studies have shown that *A. fumigatus* can acquire resistance to olorofim when exposed *in vitro* or to the recently approved agrochemical fungicide DHODH-inhibitor ipflufenoquin (7, 8). This is highly concerning and warrants continuous monitoring of the *in vitro* activity of olorofim.

We therefore continued our previous research by studying first the *in vitro* activity of olorofim against a 4-year nationwide collection of contemporary molds and dermatophytes, and second, performing a larger comparison of the serial dilution versus the ISO standard 20776-1 dilution schemes with the EUCAST methodology.

## MATERIALS AND METHODS

### Isolates

Overall, 3,550 isolates, referred to Statens Serum Institut during 2020–2023, were tested in this study. All isolates were identified with morphology, MALDI-TOF MS, and/or sequencing of ITS, β-tubulin (for *Aspergillus* spp.), and the translation elongation factor (for *Fusarium* spp.) (9). *A. fumigatus sensu stricto* (ss) was also verified with thermotolerance testing. Isolates were tested as part of the routine susceptibility testing using different batches of microtiter plates. Inocula were prepared in water with 0.1% Tween 20 from yeast glucose agar supplemented with chloramphenicol (yeast glucose chloramphenicol [YGC] agar plates, Oxoid, Thermo Fisher) and adjusted to 0.5 McFarland using an OD-meter. During the years 2020–2022, only the serial dilution method was used. The ISO standard 20776-1 dilution scheme was implemented during 2023, and the MICs for 2023 are therefore partially from serial dilution plates and partially from ISO standard 20776-1 dilution scheme plates, whereas the MICs for 2020–2022 were only from serial dilutions. Isolates tested in serial dilution plates were not retested in ISO standard 20776-1 dilution scheme plates.

### Antifungal susceptibility testing

The EUCAST E.Def 9.4 and E.Def 11.0 protocols were used for MIC determination of the following antifungal drugs olorofim (0.001–1 mg/L), amphotericin B (0.004–4 mg/L), voriconazole (0.004–4 mg/L), isavuconazole (2020 initially: 0.016–16; rest of 2020–2024: 0.008–8 mg/L), itraconazole (2020 initially: 0.016–16 mg/L, rest of 2020–2024: 0.004–4 mg/L), posaconazole (0.004–4 mg/L), and terbinafine (0.004–4 mg/L except for superficial samples: 0.03–4 mg/L followed by 0.06–8 mg/L during 2021). Pure antifungal substance stock solutions were prepared in dimethyl sulfoxide (5,000 mg/L; Merck [previously Sigma-Aldrich, Søborg Denmark]) with amphotericin B, itraconazole, voriconazole, and terbinafine (Merck); posaconazole (MSD, Ballerup, Denmark); and olorofim (F2G, Manchester, UK) and stored in aliquots at −80°C. Plates (Thermo Scientific Nunc MicroWell 96-Well, Nunclon Delta-Treated, Flat-Bottom Microplate, Fisher Scientific Biotech Line ApS, Roskilde, Denmark) were prepared either using the serial dilution directly in the medium (Serial, 2020–2023) or following the ISO standard 20776-1 with predilutions in DMSO at 200× final concentration before a 100-fold dilution in RPMI medium (RPMI 1640, buffered with MOPS and supplemented with glucose, SSI Diagnostica, Hillerød, Denmark) and transfer to the plate (ISO standard 20776-1, introduced during 2023) (10). Most isolates (*n* = 2,835) were tested using the serial dilution scheme, whereas 715 isolates were tested using the ISO standard 20776-1 dilution scheme. For *A. fumigatus*, EUCAST E.Def 10.2 was used for azole susceptibility screening until October 2022 (VIPcheck, Mediaproducts BV, Groningen, The Netherlands), whereafter MICs for voriconazole and itraconazole were determined according to EUCAST E.Def 9.4 and used as markers for isavuconazole and posaconazole susceptibility, respectively. All azole non-susceptible *A. fumigatus* were subjected to full MIC determination using EUCAST E.Def 9.4 during the entire time period. For *Microsporum*, a non-microconidia forming dermatophyte for which EUCAST has not established a reference MIC test method yet, MICs were read visually as for molds (≥90% inhibition endpoint). For quality control (QC), ATCC 204304 *A. flavus*, ATCC 204305 *A. fumigatus*, ATCC 22019 *Candida parapsilosis,* and ATCC 6258 *Candida krusei* were used as controls for the microtiter plates (data in Table S1), and *A. fumigatus* ATCC 204305, SSI-4524, and SSI-5586 were used as controls for the VIPcheck plates.

### Sequencing

*Cyp51A*, and in most cases for azole-resistant isolates not harboring a *Cyp51A* mutation, also *Hmg1* sequencing was performed for azole-resistant *A. fumigatus* and *A. terreus*. PCR amplification of *PyrE* was accomplished with the touchdown-based PCR cycling method using PCR primers AFDseq-F2 and AFDseq-R2 as previously described (5).

### Analysis

Modal MIC, geometric mean (GM), MIC_50_, and MIC_90_ were determined for species with ≥10 isolates. Wild-type upper limits (WT-ULs) were determined for species with ≥15 isolates using the ECOFFinder program as the MIC value rounded up to the nearest twofold dilution that encompasses 99.5% of WT isolates.

## RESULTS

Of the 3,550 clinical isolates, 94 (2.7%) were Mucorales, and thus inherently resistant to olorofim, and 46 (1.3%) were of doubtful clinical relevance (Table S1). Thus, 3,410 isolates were regarded as clinically relevant and potential targets for olorofim, and for these, the results are presented in detail below.

### Serial vs ISO standard 20776-1 dilution scheme

The MICs against QC strains for each dilution method were within the EUCAST target ranges for comparator drugs for the majority of drug-bug combinations. However, one dilution higher modal and GM MICs were found for the serial compared to the ISO standard 20776-1 method for olorofim and the two *Aspergillus* QC strains (Table S2). Comparative MIC data against clinical isolates for the serial dilution scheme used 2020–2023 and the ISO standard 20776-1 dilution scheme introduced in 2023 are shown in Table 1 for *Aspergillus* spp. and olorofim. The modal MICs for olorofim were again one to two dilutions higher with serial dilution compared to the ISO standard 20776-1 dilution scheme. The same phenomenon was found for olorofim MICs against the other species represented with ≥ 10 isolates (Table 2). A similar trend was found for posaconazole and voriconazole but not for amphotericin B, isavuconazole, itraconazole, and terbinafine (Tables 3 and 4). These differences resulted in one to two dilution differences in the upper MIC limit of the wild-type population (WT-UL).

**TABLE 1:** Olorofim MICs (mg/L) for *Aspergillus* spp.^*a*^^,^^*b*^^,^^*c*^

Species	Dilution method	*n*	*n* of isolates with the following MICs											MIC_50_	MIC_90_	GM-MIC	WT-UL_99.5%_^*d*^
			0.002	0.004	0.008	0.016	0.03	0.06	0.125	0.25	0.5	1	>1				
*A. flavus* SC^*e*^	ISO	22			4	**13**	4	1						0.016	0.03	0.017	0.03
	Serial	75				4	**42**	29						0.03	0.06	0.038	0.125
*A. fumigatus* SC	ISO	2				1		1						ND	ND	ND	ND
	Serial	14				2	6	5		1				ND	ND	ND	ND
*A. fumigatus ss^f^*	ISO	455			7	137	**255**	53	3					0.03	0.06	0.026	0.06
	Serial	2,047				15	450	**1,320**	254	7	1			0.06	0.125	0.056	0.125
*A. nidulans* SC	ISO	9				3	4	2						ND	ND	ND	ND
	Serial	32						**21**	11					0.06	0.125	0.077	0.125
*A. versicolor* SC	ISO	7		2	4	1								ND	ND	ND	ND
	Serial	2				1	1							ND	ND	ND	ND
*A. sydowii ss*	ISO	4	1	3										ND	ND	ND	ND
	Serial	11			3	**5**	3							0.016	0.03	0,016	ND
*A. niger* SC	ISO	27		1^*i*^		3	**13**	8	2					0.03	0.06	0.035	0.125
	Serial	131					4	48	**65**	13	1			0.125	0.25	0.099	0.25
*A. tubingensis ss*	ISO	35				2	**20**	10	3					0.03	0.06	0.040	0.125
	Serial	87				1		21	**58**	7				0.125	0.25	0.108	0.25
*A. terreus* SC	ISO	1				1								ND	ND	ND	ND
	Serial	26				3	9	**10**	4					0.06	0.125	0.045	0.25
*A. terreus ss*	ISO	12			**7**	5								0.008	0.016	0.011	0.016
	Serial	39		1^*i*^		11	**22**	5						0.03	0.06	0.026	0.06
Other, low MIC^*g*^	ISO	6			1		4	1						ND	ND	ND	ND
	Serial	11					3	6	2					ND	ND	ND	ND
Other, high MIC^*h*^	ISO	6							1			1	4	ND	ND	ND	ND
	Serial	14								3	3	1	7	ND	ND	ND	ND

^*a*^ The modal MIC is highlighted in bold.

^*b*^ ISO: ISO standard 20776-1; ND: not determined because of low number of isolates.

^*c*^ The modal MIC is given for species with ≥10 isolates.

^*d*^ The wild-type upper limit (WT-UL) is calculated for species with ≥15 isolates.

^*e*^ SC: species complex.

^*f*^ *ss*: *sensu stricto*. ID determined by BTUB sequencing, MALDI-TOF following in house method validation by BTUB sequencing or, for *A. fumigatus* specifically, thermotolerance test.

^*g*^ Low MIC species: *A. circumdati* SC (*n* = 15, with one at 0.008 mg/L), *A. candidus* SC (*n* = 1), and *A. cremei* SC (*n* = 1).

^*h*^ High MIC species: *A. ustus* SC (*n* = 9, with one at 0.125 mg/L) and *Aspergillus* section *Aspergillus* (*n* = 11).

^*i*^ Outliers are *A. neoniger* and *A. terreus ss*, both confirmed by sequencing.

**TABLE 2:** Olorofim MICs (mg/L) for other species^*a*^^,^^*b*^^,^^*c*^

Species	Dilution method	*n*	Number of isolates with the following MICs												MIC_50_	MIC_90_	GM-MIC	WT-UL_99.5%_^*d*^
			≤0.001	0.002	0.004	0.008	0.016	0.03	0.06	0.125	0.25	0.5	1	>1				
Dermatophytes																		
*Trichophyton indotineae*	ISO	12			**8**	3	1								0.004	0.008	ND	ND
	Serial	30				3	**18**	5	4						0.016	0.06	0.020	0.03
*Trichophyton interdigitale*	ISO	3			3										ND	ND	ND	ND
	Serial	14				4	**7**	1	2						0.016	0.06	0.017	ND
*Trichophyton rubrum*	ISO	54		10	**21**	15	6	2							0.004	0.016	0.005	0.03
	Serial	115			1	18	**56**	35	5						0.016	0.03	0.018	0.06
*Trichophyton violaceum*	ISO	12				2	**6**	4							0.016	0.03	0.018	ND
	Serial	4					1	2	1						ND	ND	ND	ND
Other *Trichophyton* spp.^*e*^	ISO	5			4	1									ND	ND	ND	ND
	Serial	10				1	2	**6**	1						ND	ND	ND	ND
*Microsporum audouinii*	Serial	15							2	**8**	2	1	2		0.125	1	0.18	0.25
*Microsporum canis*	ISO	5				1	2	1		1					ND	ND	ND	ND
	Serial	2						2							ND	ND	ND	ND
*Fusarium*																		
*Fusarium*, MIC ≤ 0.5 mg/L^*f*^	ISO	4					2		1			1			ND	ND	ND	ND
	Serial	9						1	1	1	2	4			ND	ND	ND	ND
*Fusarium*, MIC > 0.5 mg/L^*g*^	ISO	8											1	7	ND	ND	ND	ND
	Serial	12											2	10	ND	ND	ND	ND
Rare molds																		
*Scedosporium* spp.^*h*^	ISO	4				2	1	1							ND	ND	ND	ND
	Serial	15					1	5	4	5					ND	ND	ND	ND
*Rasamsonia piperina*	ISO	1	1												ND	ND	ND	ND
*Microascus cirrosus*	Serial	1									1				ND	ND	ND	ND

^*a*^ The modal MIC is highlighted in bold.

^*b*^ GM-MIC: geometric mean MIC; ISO: ISO standard 20776-1; ND: not determined because of the low number of isolates.

^*c*^ The modal MIC is given for species with ≥10 isolates.

^*d*^ The wild-type upper limit (WT-UL) is calculated for species with ≥15 isolates.

^*e*^ *T. benhamiae* (*n* = 5), *T. mentagrophytes* (*n* = 4), *T. soudanese* (*n* = 1), and *T. tonsurans* (*n* = 5).

^*f*^ *F. fujikuroi* SC (*n* = 9), and *F. oxysporum* SC (*n* = 4).

^*g*^ *F. tricinctum* SC (*n* = 1), *F. dimerum* SC (*n* = 9), *F. solani* SC (*n* = 9), and *F. sambucinum* SC (*n* = 1).

^*h*^ *S. apiospermum* (*n* = 14), *S. boydii* (*n* = 3), *S. dehoogii* (*n* = 1), and *Scedosporium* spp. (*n* = 1).

**TABLE 3:** MICs (mg/L) of olorofim and comparators against *Aspergillus* spp.^*a*^^,^^*b*^

Species	Dilution method (*N* isolates)	OLO	AMB	ISA		VRC		ITR		PSC		TRB
		Modal MIC^*c*^ (range) mg/L	Modal MIC (range) mg/L	Modal MIC (range) mg/L	% non-WT/R	Modal MIC (range) mg/L	% non-WT/R	Modal MIC (range) mg/L	% non-WT/R	Modal MIC (range) mg/L	% non-WT/R	Modal MIC (range) mg/L
*A. flavus* SC^*d*^	ISO (22)	**0.016 (0.008–0.06**)	1 (1–4)	1 (0.5–2)	0	**0.5 (0.5–2**)	0	0.125 (0.06–0.25)	0	**0.06 (0.06–0.25**)	0	**0.25 (0.06–2**)
	Serial (75)	**0.03 (0.016–0.06**)	1 (0.25–4)	1 (0.25–2)	0	**1 (0.25–2**)	0	0.125 (0.03–0.5)	0	**0.125 (0.06–0.5**)	0	**0.125 (0.03–1**)
*A. fumigatus* SC	ISO (2)	ND (0.016–0.06)	ND (0.5–2)	ND (1–2)	NA	ND (4)	NA	ND (0.5–1)	NA	ND (0.25)	NA	ND (0.5–1)
	Serial (14)	ND (0.016–0.25)	ND (0.125 to >4)	ND (0.125–4)	NA	ND (0.125 to >4)	NA	ND (0.008 to >4)	NA	ND (0.016–0.5)	NA	ND (0.125–1)
*A. fumigatus ss^e^*	ISO (455)	**0.03 (0.008–0.125**)	0.5 (0.125–1)	1 (0.06 to >8)^*f*^	4.8	**0.5 (0.125 to >4)**	5.1	0.25 (0.03 to >4)	5.3	**0.06 (0.008 to >4)**	5.3	>4 (1 to >4)
	Serial (2047)	**0.06 (0.016–0.5**)	0.5 (0.016–2)^*j*^	1 (0.25 to >8)	7.1	**1 (0.06 to >4)**	7.2	0.25 (0.03 to >4)	7.1	**0.125 (0.03 to >4)**	7.5	>4 (0.5 to >4)
*A. nidulans* SC	ISO (9)	ND (0.016–0.06)	ND (0.25–4)	ND (0.125–0.25)	0	ND (0.25–0.5)	0	ND (0.06–0.25)	0	ND (0.06–0.25)	0	0.125–0.5
	Serial (32)	0.06 (0.06–0.125)	1 (0.25–4)	0.25 (0.125–0.5)	15.6	0.5 (0.125–0.5)	0	0.25 (0.06–1)	0	0.25 (0.06–0.25)	0	0.5 (0.125–0.5)
*A. versicolor* SC	ISO (7)	ND (0.004–0.016)	ND (0.25–4)	ND (0.25–1)	NA	ND (0.25–1)	NA	ND (0.125–0.25)	NA	ND (0.06–0.25)	NA	ND (0.06–1)
	Serial (2)	ND (0.016–0.03)	ND (1, 2)	ND (0.25–0.5)	NA	ND (0.5–1)	NA	ND (0.125 to >4)	NA	ND (0.06–0.5)	NA	ND (0.06–0.25)
*A. sydowii ss*	ISO (4)	ND (0.002–0.004)	ND (0.5–1)	ND (0.5)	NA	ND (1)	NA	ND (0.25–0.5)	NA	ND (0.125–0.25)	NA	ND (0.06–0.125)
	Serial (11)	0.016 (0.008–0.03)	1 (0.25–4)	1 (0.25–2)	NA	0.5 (0.5–2)	NA	0.25–0.5 (0.125–1)	NA	0.25 (0.125–0.5)	NA	0.125/0.25 (0.06–0.25)
*A. niger* SC	ISO (27)	**0.03 (0.004–0.125**)	0.25 (0.03-0.5)	**1 (0.25–4**)	0	**0.5 (0.125–2**)	0	**0.25 (0.03–1**)	0	**0.125 (0.016–0.5**)	0	0.5 (0.06–1)
	Serial (131)	**0.125 (0.03–0.5**)	0.25 (0.125–1)	**2 (0.25 to >8**)	4.6	**1 (0.25 to >4**)	3.1	**0.5 (0.125 to >4**)	5.3	**0.25 (0.06–1**)	1.5	0.5 (0.125–4)
*A. tubingensis ss^g^*	ISO (35)	**0.03 (0.016–0.125**)	**0.125 (0.06–0.5**)	**1 (0.25–4**)	0	1 (0.25–2)	0	0.5 (0.125 to >4)	2.9	**0.125 (0.03–0.5**)	0	0.5 (0.125–2)
	Serial (87)	**0.125 (0.016–0.25**)	**0.25 (0.06–0.5**)	**2 (0.5 to > 8**)	8.0	1 (0.25 to >4)	2.3	0.5 (0.125 to >4)	9.2	**0.25 (0.06–1**)	1.2	0.5 (0.25–1)
*A. terreus* SC	ISO (1)	ND (0.016)	ND (4)	ND (2)	100	ND (2)	0	ND (1)	0	ND (0.5)	100	0.25
	Serial (26)	0.06 (0.016–0.125)	2 (1 to >4)^*h*^	2 (1 to >8)	88.5	2 (0.5 to >4)	26.9	>4 (0.125 to >4)	69.2	0.5 (0.06–1)	73.1	0.5 (0.25–1)
*A. terreus ss*	ISO (12)	**0.008 (0.008–0.016**)	2 (2–4)	1 (1–8)	58.3	1 (1–4)	25	**1 (0.125 to >4**)	8.3	**0.125 (0.125–0.5**)	25	0.25 (0.125–1)
	Serial (39)	**0.03 (0.004–0.06**)	2 (1 to >4)^*h*^	1 (0.25 to >8)	59.0	1 (0.25 to >4)	23.1	**>4 (0.06 to >4**)	41.0	**0.5 (0.03–1**)	51.3	0.25 (0.125–1)
Other *Aspergillus*^*i*^	ISO (12)	NA (0.008 to >1)	NA (0.06 to >4)	NA (0.06–8)	NA	NA (0.125 to >4)	NA	NA (0.016 to >4)	NA	NA (0.008 to >4)	NA	NA (0.016–1)
	Serial (25)	NA (0.03 to >1)	NA (0.03 to >4)	NA (0.06–8)	NA	NA (0.06 to >4)	NA	NA (0.016 to >4)	NA	NA (0.03 to >8)	NA	NA (0.06–1)

^*a*^ Species-drug pairs with different modal MIC between ISO standard 20776-1 (ISO) and Serial dilutions are in bold.

^*b*^ OLO: olorofim; AMB: amphotericin B; ISA: isavuconazole; VRC: voriconazole; ITR: itraconazole; PSC: posaconazole; TRB: terbinafine. NA: not applicable because of absence of breakpoints or isolates from different species; ND: not determined because of low number of isolates.

^*c*^ The modal MIC is given for species with ≥10 isolates.

^*d*^ SC: species complex.

^*e*^ *ss*: *sensu stricto*. ID determined by BTUB sequencing, MALDI-TOF following in house method validation by BTUB sequencing or, for *A. fumigatus* specifically, thermotolerance test.

^*f*^ For *A. fumigatus ss*, azole MICs are for the subset of isolates subjected to full MIC determination and indicated by underscoring.

^*g*^ Azole susceptibility categorization of *A. tubingensis* by *A. niger* ECOFFs/breakpoints.

^*h*^ The highest concentration of AMB is below the ECOFF and the no. of AMB non-WT isolates was not estimated.

^*i*^ *Aspergillus* section *Aspergillus* (11), *A. candidus* SC (1), *A. circumdati* SC (15), *A. cremei* SC (1), and *A. ustus* SC (9).

^*j*^ Six *A. fumigatus ss* with AMB = 2 mg/L corresponding to 0.3%.

**TABLE 4:** MICs (mg/L) of olorofim and comparators against other molds as range and modal MIC^*a*^^,^^*b*^

Species	Dilution method (*N* isolates)	Olo	AMB	ISA	VRC		ITR		PSC	TRB	
		Modal MIC^*c*^ (range) mg/L	Modal MIC (range) mg/L	Modal MIC (range) mg/L	Modal MIC (range) mg/L	% non-WT/R	Modal MIC (range) mg/L	% non-WT/R	Modal MIC (range) mg/L	Modal MIC (range) mg/L	% non-WT/R
Dermatophytes											
*T. indotineae*	ISO (12)	**0.004 (0.004–0.016**)	1 (0.5–1)	**0.06 (0.03–4**)	**0.06 (0.03–2**)	8.3	0.016 (≤0.004–0.5)	8.3	**0.008 (≤0.004–0.25**)	>4 (0.06 to >4)	91.7
	Serial (29–30)	**0.016 (0.008–0.06**)	1 (0.5 to >4)	**0.25 (0.06–2**)	**0.125 (0.03–1**)	0	0.016 (≤0.004–0.25)	0	**0.03 (0.008–0.25**)	>4 (0.03 to >4)	90.0
*T. interdigitale*	ISO (3)	NA (0.004)	NA (0.5–1)	NA (≤0.008–0.06)	NA (0.03–0.125)	NA	NA (0.008–0.03)	NA	NA (≤0.004–0.016)	NA (≤0.004–0.03)	NA
	Serial (14)	0.016 (0.008–0.06)	1 (0.5 to >4)	0.06 (0.016–0.5)	0.125 (0.03–0.5)	NA	0.03 (0.008–0.125)	NA	0.03 (0.016–0.06)	0.016 (≤0.004–0.5)	NA
*T. rubrum*	ISO (53–54)	**0.004 (0.002–0.03**)	1 (0.125–4)	**0.03 (≤0.008–0.125**)	**0.06 (0.016–0.5**)	1.9	**0.06 (0.016–0.25**)	0	**0.03 (≤0.004–0.125**)	0.016 (≤0.004 to >4)	40.7
	Serial (114–115)	**0.016 (0.004–0.06**)	1 (0.125 to >4)	**0.06 (≤0.008–0.25**)	**0.125 (0.008–1**)	14.0	**0.125 (0.008–4**)	4.3	**0.06 (0.008–0.5**)	0.016 (≤0.004 to >4)	48.7
*T. violaceum*	ISO (11, 12)	0.016 (0.008–0.03)	1 (0.5 to >4)	0.06 (≤0.008–0.125)	1 (0.06–2)	NA	0.25 (≤0.004–2)	NA	0.25 (0.008–0.25)	0.03–0.125 (0.008–0.125)	NA
	Serial (114–115)	NA (0.016–0.06)	NA (0.5–1)	NA (0.016–0.125)	(0.03–2)	NA	NA (0.016–0.125)	NA	NA (0.06–0.125)	NA (0.008–0.03)	NA
Other *Trichophyton^d^*	ISO (5)	NA (0.004–0.008)	NA (0.5–4)	NA (≤0.008–0.125)	NA (0.016–0.125)	NA	NA (≤0.004–0.25)	NA	NA (≤0.004–0.125)	NA (≤0.004–0.016)	NA
	Serial (10)	NA (0.008–0.06)	NA (0.5–2)	NA (≤0.008–0.5)	NA (0.03–0.5)	NA	NA (≤0.004–0.125)	NA	NA (0.008–0.125)	NA (0.008–0.03)	NA
*Microsporum* *audouinii*	Serial (13–15)	0.125 (0.06–1)	0.25 (0.06–1)	0.125 (0.125–1)	1 (0.06–2)	NA	0.125/1 (0.06 to > 4)	NA	0.25/0.5 (0.125–2)	0.06 (0.03–0.125)	NA
*Microsporum* *canis*	ISO (5)	NA (0.008–0.125)	NA (0.25–1)	NA (0.06–0.25)	NA (0.125–0.25)	NA	NA (0.125–1)	NA	NA (0.125–1)	NA (0.06–0.5)	NA
	Serial (2)	NA (0.03)	NA (0.125)	NA (0.06–0.125)	NA (0.125)	NA	NA (0.125–0.5)	NA	NA (0.125–0.25)	NA (0.06–0.125)	NA
*Fusarium*											
*Fusarium*, all^*e*^	ISO (12)	NA (0.016 to >1)	NA (0.25–1)	NA (8 to >8)	NA (2 to >4)	NA	NA (>4)	NA	NA (1 to >4)	NA (1 to >4)	NA
	Serial (21)	NA (0.03 to >1)	NA (0.5–2)	NA (4 to >8)	NA (2 to >4)	NA	NA (>4)	NA	NA (0.25 to >4)	NA (0.5 to >4)	NA
Rare molds											
*Scedosporium* spp.^*f*^	ISO (4)	NA (0.008–0.03)	NA (2–4)	NA (0.5–8)	NA (0.125–1)	NA	NA (0.25 to >4)	NA	NA (0.25–1)	NA (>4)	NA
	Serial (15)	NA (0.016–0.125)	NA (0.125 to >4)	NA (1 to >8)	NA (0.25–2)	NA	NA (0.25 to >4)	NA	NA (0.25–2)	NA (>4)	NA
Other rare molds^*g*^	ISO (1)	NA (≤0.001)	NA (0.5)	NA (>8)	NA (>4)	NA	NA (0.5)	NA	NA (0.25)	NA (1)	NA
	Serial (1)	NA (0.25)	NA (>4)	NA (4)	NA (4)	NA	NA (>4)	NA	NA (>4)	NA (>4)	NA

^*a*^ Species-drug pairs with different modal MIC between ISO standard 20776-1 (ISO) and Serial dilutions are in bold.

^*b*^ OLO: olorofim; AMB: amphotericin B; ISA: isavuconazole; VRC: voriconazole; ITR: itraconazole; PSC: posaconazole; TRB: terbinafine; NA: not applicable.

^*c*^ Modal MIC for species with ≥10 isolates.

^*d*^ *T. benhamiae* (5), *T. mentagrophytes* (4), *T. soudanese* (1), and *T. tonsurans* (5).

^*e*^ *F. fujikuroi* SC (9), *F. oxysporum* SC (4), *F. tricinctum* SC (1), *F. dimerum* SC (9), *F. solani* SC (9), and *F. sambucinum* SC (1).

^*f*^ *S. apiospermum* (14), *S. boydii* (3), *S. dehoogii* (1), and *Scedosporium* spp. (1).

^*g*^ *Rasamsonia piperina* (ISO) and *Microascus cirrosus* (serial).

### Species-specific susceptibility

Species-specific olorofim MIC ranges and modal MICs were comparable across the four years (identical or within one dilution step) for the most prevalent *Aspergillus* species and are presented in Table 1 and Tables S3 and S4 in alphabetical order. The agreement between olorofim MICs for Danish isolates from 2020 to 2023 and the previously reported time periods 2016–2017 and 2018–2019, respectively, were high (6). Uniform Gaussian MIC distributions spanning ≤5 dilutions were seen for *A. fumigatus sensu stricto* and all *Aspergillus* complexes with ≥10 isolates. For section *Nidulantes* isolates, a differential MIC pattern was seen for *A. nidulans* complex and *A. versicolor* complex isolates. Of note, high olorofim MICs (>1 mg/L) were found against section *Aspergillus* isolates (two *A. pseudoglaucus*, one *A. chevalieri*, two *A. intermedius*, and one *A. montevidensis*). Most *Aspergillus* spp. had WT-UL_99.5%_ between 0.03 and 0.125 mg/L with the ISO standard 20776-1 method, except *A. terreus sensu stricto,* for which WT-UL_99.5%_ was lower (0.016 mg/L), although few isolates were tested.

### Azole non-susceptible *Aspergillus*

Olorofim susceptibility was not affected in 165 azole-resistant *A. fumigatus* isolates, of which 96 harbored 23 unique Cyp51A alterations (including six of environmental origin), and seven harbored Hmg1 alterations: F262del (*n* = 1), H237Y (*n* = 1), and L289P (*n* = 5). Similarly, olorofim susceptibility was not affected in 37 *A*. *terreus* isolates harboring the following Cyp51A substitutions: G51A (*n* = 21), M217I (*n* = 15), and V168I/Q277R/V486E (*n* = 1) (Tables 5 and 6).

**TABLE 5:** Olorofim MIC data (mg/L) for *Aspergillus* isolates with and without *Cyp51A* and *Hmg1* alterations conferring azole-resistance^*a*^^,^^*b*^

Species group	*n*	Dilution	Olorofim MIC (mg/L)							
		Method	0.004	0.008	0.016	0.03	0.06	0.125	0.25	0.5
*A. fumigatus*										
*Cyp51A* alterations	141	Serial			2	15	**96**	27	1	
	24	ISO			12	7	**3**	2		
*Hmg1*	7				3		2	1		1
WT	34	Both		1	1	5	**19**	7		1
Not sequenced	2,303	Both		6	137	678	**1,255**	221	6	
In total	2,509	Both		7	155	705	**1,375**	258	7	2
*A. terreus SC*										
*Cyp51A* alterations	30	Serial			2	**17**	9	2		
	7	ISO		3	4					
Not sequenced	34	Both	1	4	14	**13**	6	2		
In total	77	Both	1	7	20	**30**	15	4		

^*a*^ Modal MICs are shown in bold.

^*b*^ ISO: ISO standard 20776-1 (ISO).

**TABLE 6:** Details of isolates with olorofim MIC above the WT-UL (>0.125 mg/L for *A. fumigatus*, and >0.06 mg/L for *A. terreus*)^*b*^

Species	Gender (F/M)	Age (years)	Source	Alteration		MIC (mg/L)						
				CYP51A	PyrE	OLO	AMB	ISA	VRC	ITR	PSC	TRB
*A. fumigatus*	F	56	Tracheal aspirate	WT	WT	0.25	1	1	S	S	S	ND
*A. fumigatus*	F	12	Sputum	WT	WT	0.25	0.25	1	1	0.5	0.125	>4
*A. fumigatus*	F	26	Sputum	WT	WT	0.25	0.5	0.5	S	S	S	ND
*A. fumigatus*	F	69	Sputum	WT	Q35L^*a*^	0.25	0.5	1	0.5	0.25	0.06	4
*A. fumigatus*	F	59	BAL	WT	WT	0.5	0.25	1	0.25	0.125	0.06	>4
*A. fumigatus*	M	51	Ear	TR_34_/L98H	WT	0.25	0.25	8	>4	>4	0.5	4
*A. terreus* SC	F	43	Sputum	M217I	WT	0.125	>4	>4	4	>4	0.5	0.5
*A. terreus* SC	M	13	Sputum	G51A	WT	0.125	2	1	2	>4	0.5	1
*A. terreus* SC	F	44	Sputum	M217I	WT	0.125	2	>8	>4	>4	0.5	0.5
*A. terreus* SC	M	14	Sputum	G51A	WT	0.125	2	2	1	>4	1	1

^*a*^ This mutation is outside the hotspot area and probably does not confer resistance. SC: species complex.

^*b*^ OLO: olorofim, AMB: amphotericin B, ISA: isavuconazole, VRC: voriconazole, ITR: itraconazole, PSC: posaconazole, and TRB: terbinafine.

### Rare molds

Olorofim activity varied across *Fusarium* spp. (Table 2). Isolates of *Fusarium fujikuroi* SC were the most susceptible (*Fusarium proliferatum, Fusarium musae*), whereas MICs against *Fusarium solani* SC, *Fusarium dimerum*, *Fusarium tricinctum*, and *Fusarium sambucinum* were consistently ≥1 mg/L. *Scedosporium* was as susceptible as *A. fumigatus* with MICs ≤ 0.125 mg/L (Table 2; Table S3). Low MICs (≤0.03 mg/L) were found for *Talaromyces* spp., whereas *Penicillium* and *Paecilomyces* spp. were less susceptible (modal MICs 0.125 and 0.5 mg/L, respectively), and some other rare genera like *Acremonium* (*n* = 1) and *Scopulariopsis* (*n* = 1) with MICs > 1 mg/L (Table S1).

### Dermatophytes

Uniform olorofim MIC ranges were found for all *Trichophyton* (*T*.) species. Most MICs were ≤0.06 mg/L and modal MICs were 0.016–0.03 mg/L across dermatophyte species except *Microsporum audouinii,* for which the modal MIC was 0.125 mg/L (Table 2).

### Changes over the years

**Fig 1:**
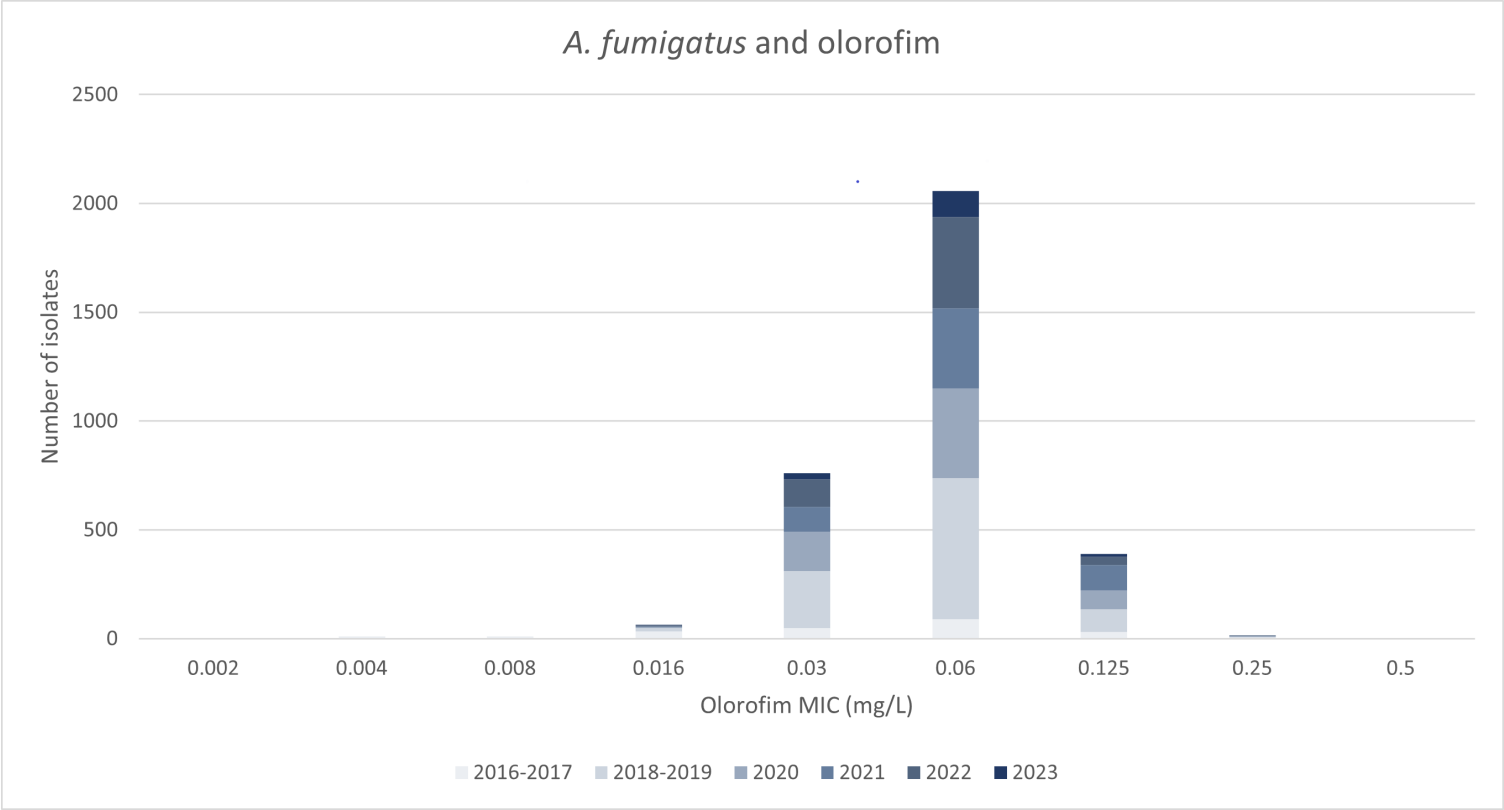
Olorofim MIC data for *A. fumigatus sensu stricto* over the years with serial dilution scheme.

The annual modal olorofim MIC for *A. fumigatus* did not change over the years 2020–2023 (Fig. 1). Compared with the MICs against clinical isolates from 2016 to 2019 (5, 6), the species-specific modal MICs were the same for most species. Exceptions were (i) *A. niger* SC, for which the modal MIC increased by one dilution from 0.06 to 0.125 mg/L, and (ii) *T. rubrum,* for which the modal MIC decreased by two dilutions from 0.06 to 0.016–0.03 mg/L upon the transition from the use of the reference method for molds to the new dermatophyte reference method.

## DISCUSSION

Olorofim displayed broad-spectrum activity against most *Aspergillus*, *Scedosporium,* and dermatophyte species in this study. For *Aspergillus*, exceptions were *Aspergillus ustus* (modal MIC 0.25–0.5 mg/L) and section *Aspergillus* isolates (modal MIC >1 mg/L). In comparison, *in vitro* susceptibility of *Fusarium* was lower and more diverse. Most susceptible were *F. fujikuroi* SC and *Fusarium oxysporum* SC (MICs 0.125–1 mg/L), whereas most MICs for *F. dimerum* and *F. solani* SC isolates were high (>1 mg/L). Finally, no activity was found against Mucorales, in agreement with previous findings and the fact that Mucorales possess class 1A DHODH and not class 2 DHODH enzymes, which are the target for olorofim (4).

Olorofim MICs against dermatophytes were low (modal MIC ≤0.125 mg/L). Given the global increase in terbinafine resistance and the availability of an oral option, the role of olorofim in *Trichophyton* infection should be further explored (11). The differential activity observed against *Trichophyton* and *Microsporum* can, at least in part, be explained by technical reasons. A less strict endpoint is adopted for MIC determination of conidia-forming dermatophytes (≥50% growth inhibition) compared to that used for molds and *Microsporum* (≥90% inhibition) (12–14). This results in a one to two twofold dilution difference in the MICs.

Regarding rare mold species, although few isolates were tested, low MICs were found for *Talaromyces* spp., somewhat higher MICs were found against *Penicillium* and *Paecilomyces* spp. (modal 0.125 and 0.5 mg/L, respectively), while *Acremonium* and *Scopulariopsis* were the least susceptible genera with MICs > 1 mg/L.

The WT-ULs for species with >15 isolates were 0.125–0.25 mg/L for most *Aspergillus* spp., *Scedosporium*, and *M. audouinii,* whereas for other dermatophytes, WT-UL values were lower (0.03–0.06 mg/L). As expected, *Cyp51A* and *Hmg1* alterations did not affect olorofim MICs against *A. fumigatus* and *A. terreus*. Of note, *PyrE* mutations were not found for *A. fumigatus* isolates with higher MICs (0.125–0.5 mg/L) except one isolate which harboured a Q35L alteration (Table 6). This residue is located in the variable N-terminus of the protein in the predicted mitochondrial target sequence, away from the hotspot area of G119 and is as such likely without importance for resistance in the isolate (7, 15).

*In vitro* acquisition of olorofim resistance after exposure to olorofim or to ipflufenoquin has been described (7, 8). We found no significant differences in olorofim susceptibility for *A. fumigatus* over the years 2020–2023 or when comparing to our previous study covering 2016–2017 (6) and 2018–2019 (5, 7). This is expected, since olorofim is not yet in clinical use, and ipflufenoquin is only recently approved for use in selected countries outside Europe. However, continued monitoring of susceptibility is warranted.

A technical variable of *in vitro* susceptibility testing that seems to affect olorofim MICs is the dilution scheme followed for microplate preparation. Serial dilution in the medium resulted in higher MICs compared to those obtained for the ISO standard 20776-1 standard scheme. This shift in the MIC distributions had an impact on the WT-ULs, which, for example for *A. fumigatus*, were higher with the serial dilution (0.125 mg/L) compared to the ISO standard 20776-1 standard (0.06 mg/L). This phenomenon was observed across species and also for posaconazole and voriconazole and particularly impacted the species for which the modal MIC was low (<0.25 mg/L). Previous systematic comparisons of the serial and ISO standard 20776-1 dilution scheme for olorofim did not show such consistent differences, although only a few (*n* = 53) isolates were tested and all with the same batches of plates (6). It has previously been found that the number of pipette tip changes during serial dilution for plate preparation impacts the MIC for some agents, particularly anidulafungin, micafungin, and isavuconazole, and most significantly against low MIC species (10). However, it was also found that two pipette tip changes evenly distributed over the concentration range, which was followed in the present study, generated similar results as with the ISO standard 20776-1 method. We speculate that drug loss due to binding to plastics during the transfers of drug in aqueous solution in interim tubes and reservoirs during the longer routine plate production process for serial dilution plates may have led to higher MICs than when the ISO standard 20776-1 dilution scheme is followed. Consequently, we now use the ISO standard 20776-1 dilution method and recommend that this is followed.

In conclusion, olorofim displayed broad, potent, and stable *in vitro* activity against most molds (serial dilution MICs < 0.5 mg/L, modal MICs 0.016–0.125 mg/L) except *Aspergillus* section *Aspergillus*, *F. dimerum*, *F. solani* SC, Mucorales, and some rare molds. Technical variables may affect olorofim MIC testing, and therefore, complying with the ISO standard 20776-1 dilution method and establishment of QC ranges are important to standardize MIC testing across different laboratories. No mutations within the hotspot area of the target gene were found for the few isolates with elevated olorofim MICs. We look forward to seeing how this potent *in vitro* activity translates into clinical efficacy, which remains to be learned from the ongoing Phase 3 clinical trial.

## SUPPLEMENTAL MATERIAL

Supplemental tables aac.00353-25-s0001.docx Tables S1 to S4.

ASM does not own the copyrights to Supplemental Material that may be linked to, or accessed through, an article. The authors have granted ASM a non-exclusive, world-wide license to publish the Supplemental Material files. Please contact the corresponding author directly for reuse.
