# Supplementary material for: EUCAST olorofim MICs for 3,550 Danish mold and dermatophyte isolates from 2020 to 2023
Source: Antimicrob Agents Chemother. 2025 Jun 26;69(8):e00353-25. doi: 10.1128/aac.00353-25 (PMC12326962; doi:10.1128/aac.00353-25)
Supplement: Supplemental tables — Tables S1 to S4. [file aac.00353-25-s0001.docx]

**Supplementary table 1**. MICs (mg/L) of olorofim and comparators for rare moulds with doubtful clinical relevance in alphabetical order.

| **Species** | **Dilution method** | ***N*** | **Olorofim MIC (mg/L)** | | | | | | | | | | |  | **MIC or range for comparators (mg/L)** | | | | | |
| --- | --- | --- | --- | --- | --- | --- | --- | --- | --- | --- | --- | --- | --- | --- | --- | --- | --- | --- | --- | --- |
|  |  |  | **0.002** | **0.004** | **0.008** | **0.016** | **0.03** | **0.06** | **0.125** | **0.25** | **0.5** | **1** | **>1** |  | **AMB** | **ISA** | **VRC** | **ITR** | **PSC** | **TRB** |
| *Acremonium* spp. | Serial | 1 |  |  |  |  |  |  |  |  |  | 1 |  |  | 4 | 8 | 4 | >4 | >4 | 0.25 |
| *Acremonium sclerotigenum* | Serial | 1 |  |  |  |  |  |  |  |  |  |  | 1 |  | 2 | >8 | >4 | >4 | >4 | 0.5 |
| *Alternaria infectoria* | Serial | 1 |  |  |  |  |  |  |  |  |  |  | 1 |  | 0.125 | >8 | >4 | 1 | 0.06 | 0.25 |
| *Arthroderma curreyi* | ISO | 1 | 1 |  |  |  |  |  |  |  |  |  |  |  | 0.5 | 1 | 0.5 | 0.25 | 0.125 | 0.25 |
| *Cephalotrichum species* | Serial | 1 |  |  |  |  | 1 |  |  |  |  |  |  |  | 1 | 8 | >4 | >4 | >4 | 2 |
| *Chrysosporium keratinophilum* | Serial | 1 |  |  |  |  |  |  | 1 |  |  |  |  |  | 1 | 1 | 0.5 | 2 | 0.5 | 0.25 |
| *Cladosporium halotolerans* | Serial | 1 |  |  |  |  |  |  |  |  |  |  | 1 |  | 0.5 | 8 | 2 | 0.25 | 0.06 | 0.5 |
| *Cladosporium species* | Serial | 1 |  |  |  |  |  |  |  |  |  |  | 1 |  | 2 | 4 | 1 | 0.5 | 0.25 | 1 |
| *Cypellophora europaea* | Serial | 1 |  |  |  |  |  |  |  |  |  | 1 |  |  | 1 | 0.5 | 0.25 | 0.016 | 0.016 | 0.25 |
| *Epidermophyton floccosum* | Serial | 1 |  |  |  |  |  | 1 |  |  |  |  |  |  | 0.5 | 0.125 | 0.125 | 0.06 | 0.06 | 0.125 |
| *Hormographiella aspergillata* | Serial | 1 |  |  |  |  |  |  |  |  |  |  | 1 |  | 0.06 | >8 | >4 | >4 | >4 | >4 |
| *Ochroconis musae* | Serial | 1 |  |  |  |  |  |  |  |  | 1 |  |  |  | >4 | >8 | >4 | 0.5 | 0.25 | 0.25 |
| *Paecilomyces formosus* | ISO | 1 |  |  |  |  |  |  |  |  |  | 1 |  |  | 0.25 | >8 | >4 | 0.125 | 0.125 | 4 |
|  | Serial | 1 |  |  |  |  |  |  |  | 1 |  |  |  |  | 0.125 | >8 | >4 | 0.06 | 0.125 | 0.5 |
| *Paecilomyces maximus* | Serial | 1 |  |  |  |  |  |  |  | 1 |  |  |  |  | 0.06 | >8 | >4 | 0.125 | 0.125 | 0.5 |
| *Paecilomyces variotii* | Serial | 7 |  |  |  |  |  |  | 6 | 1 |  |  |  |  | 0.016-0.25 | >8 | >4 | 0.03-0.125 | 0.06-0.125 | 2->4 |
| *Paecilomyces* spp. | Serial | 1 |  |  |  |  |  |  |  |  | 1 |  |  |  | 0.06 | >8 | >4 | 0.06 | 0.125 | >4 |
| *Penicillium chrysogenum* | Serial | 1 |  |  |  |  | 1 |  |  |  |  |  |  |  | 0.5 | 2 | 2 | 0.25 | 0.25 | 1 |
| *Penicillium citrinum* | Serial | 8 |  |  |  |  |  |  |  | 2 | 3 | 2 | 1 |  | 0.125-2 | 0.5->8 | >4 | 0.25->4 | 0.06-2 | 0.125-1 |
| *Penicillium hetheringgtonii* | Serial | 1 |  |  |  |  |  |  |  |  | 1 |  |  |  | 0.125 | 2 | >4 | 1 | 0.5 | 0.25 |
| *Penicillium thomii* | Serial | 1 |  |  |  |  |  |  | 1 |  |  |  |  |  | 0.5 | 2 | 2 | 0.25 | 0.06 | 1 |
| *Penicillium* spp. | ISO | 2 |  |  |  |  | 1 |  |  | 1 |  |  |  |  | 0.5-1 | 0.25-2 | 1->4 | 0.06-1 | 0.06-0.25 | 1 |
| *Phialemoniopsis curvata* | Serial | 1 |  |  |  |  |  |  |  |  | 1 |  |  |  | 1 | 0.5 | 1 | 0.125 | 0.125 | 2 |
| *Purpureocillium lilacinum* | Serial | 2 |  |  |  |  |  |  |  |  |  |  | 2 |  | >4 | 0.5 | 0.25-0.5 | 0.5->4 | 0.125-0.25 | 0.5 |
| *Schizophyllum commune* | ISO | 1 |  |  |  |  |  |  |  |  |  |  | 1 |  | 0.03 | 0.5 | 0.25 | 0.25 | 0.5 | >4 |
| *Scopulariopsis brevicaulis* | Serial | 1 |  |  |  |  |  |  |  |  |  |  | 1 |  | >4 | >8 | >4 | >4 | >4 | >4 |
| *Talaromyces amestolkiae* | Serial | 1 |  |  |  | 1 |  |  |  |  |  |  |  |  | 0.125 | >8 | >4 | >4 | >4 | 0.06 |
| *Talaromyces columbinus* | Serial | 2 |  |  | 2 |  |  |  |  |  |  |  |  |  | 1 | >8 | >4 | >4 | >4 | 2 |
| *Talaromyces* spp. | Serial | 1 |  |  |  |  | 1 |  |  |  |  |  |  |  | 0.06 | >8 | >4 | >4 | >4 | 0.25 |
| *Trichoderma* spp. | ISO | 1 |  |  | 1 |  |  |  |  |  |  |  |  |  | 0.25 | 8 | 0.5 | 1 | 2 | 0.5 |

OLO: olorofim, AMB: amphotericin B, ISA: isavuconazole, VRC: voriconazole, ITR: itraconazole, PSC: posaconazole, TRB: terbinafine. ISO: ISO standard 20776-1 (ISO).

**Supplementary table 2**. MICs (mg/L) of EUCAST control strains for 2020-2023. EUCAST recommended ranges are included for comparison.

|  |  | **Olo** | |  | **AMB** | |  | **ISA** | |  | **VOR** | |  | **ITR** | |  | **PSC** | |  | **TRB** | |
| --- | --- | --- | --- | --- | --- | --- | --- | --- | --- | --- | --- | --- | --- | --- | --- | --- | --- | --- | --- | --- | --- |
| **Strains Dilution**  **method** |  | ***N*** | **Modal MIC**  **(range)**  **GM** |  | ***N*** | **Modal MIC (range)**  **% within range** |  | ***N*** | **Modal MIC (range)**  **% within range** |  | ***N*** | **Modal MIC (range)**  **% within range** |  | ***N*** | **Modal MIC (range)**  **% within range** |  | ***N*** | **Modal MIC (range)**  **% within range** |  | ***N*** | **Modal MIC (range)**  **% within range** |
| ATCC 204304 *A. flavus* |  |  |  |  |  |  |  |  |  |  |  |  |  |  |  |  |  |  |  |  |  |
| ISO |  | 12 | 0.016  (0.016-0.03)  0.017 |  | 12 | 1  (0.5-2)  100% |  | 7 | NA^1^  (1)  NA |  | 12 | 1  (0.5-1)  100% |  | 12 | 0.125  (0.06-1)  75% |  | 7 | NA  (0.06-0.125)  57.1% |  | 7 | NA  (0.5-1)  100% |
| Serial |  | 74 | 0.03  (0.016-0.06)  0.039 |  | 91 | 1  (0.25-2)  98% |  | 73 | 1  (1-2)  NA |  | 74 | 1  (0.5-2)  100% |  | 89 | 0.125  (0.06-1)  95.5% |  | 71 | 0.125  (0.125-0.25)  100% |  | 85 | 0.5  (0.5-2)  98.8% |
| EUCAST |  |  | NA |  |  | (0.5-2) |  |  | NA |  |  | (0.5-2) |  |  | (0.125-0.5) |  |  | (0.125-0.5) |  |  | (0.25-1) |
| ATCC 204305 *A. fumigatus* |  |  |  |  |  |  |  |  |  |  |  |  |  |  |  |  |  |  |  |  |  |
| ISO |  | 16 | 0.03  (0.03-0.06)  0.031 |  | 16 | 0.5  (0.25-1)  100% |  | 5 | NA  (0.5-1)  NA |  | 16 | 1  (0.5-1)  100% |  | 16 | 0.25  (0.25-1)  93.8% |  | 5 | NA  (0.06-0.125)  100% |  | 5 | NA  (2-4)  NA |
| Serial |  | 119 | 0.06  (0.03-0.25)  0.075 |  | 126 | 0.5  (0.25-1)  100% |  | 119 | 1  (0.5-2)  NA |  | 80 | 1  (0.5-2)  85% |  | 81 | 0.25  (0.125-1)  98.8% |  | 77 | 0.125  (0.06-0.25)  100% |  | 81 | 2  (1->4)  NA |
| EUCAST |  |  | NA |  |  | (0.25-1) |  |  | NA |  |  | (0.25-1) |  |  | (0.125-0.5) |  |  | (0.03-0.25) |  |  | NA |
| ATCC 22019 *C. parapsilosis* |  |  |  |  |  |  |  |  |  |  |  |  |  |  |  |  |  |  |  |  |  |
| ISO |  | 13 | >1  (>1)  >1 |  | 38 | 0.125  (0.06-0.5)  97% |  | 7 | NA  (≤0.008-0.03)  100%^2^ |  | 38 | 0.03  (0.008-0.06)  94.7% |  | 11 | 0.06  (0.016-0.125)  72.7% |  | 6 | NA  (0.008-0.03)  83.3% |  | 6 | NA  (0.06-0.25)  NA |
| Serial |  | 61 | >1  (>1)  >1 |  | 270 | 0.25  (0.125-1)  100% |  | 62 | 0.03  (≤0.008-0.06)  82.3% |  | 190 | 0.03  (0.016-0.125)  98.9% |  | 142 | 0.06  (0.016-0.125)  99.3% |  | 61 | 0.03  (0.016-0.06)  100% |  | 82 | 0.125-0.25  (0.06-1)  NA |
| EUCAST |  |  | NA |  |  | (0.125-1) |  |  | (0.008-0.03) |  |  | (0.016-0.06) |  |  | (0.03-0.125) |  |  | (0.016-0.06) |  |  | NA |
| ATCC 6258 *C. krusei* |  |  |  |  |  |  |  |  |  |  |  |  |  |  |  |  |  |  |  |  |  |
| ISO |  | 10 | >1  (>1)  >1 |  | 30 | 0.25  (0.125-0.5)  100% |  | 4 | NA  (0.016-0.06)  100% |  | 30 | 0.125  (0.06-0.25)  100% |  | 10 | 0.03  (0.016-0.06)  80% |  | 4 | NA  (0.008-0.03)  75% |  | 4 | NA  (>4)  NA |
| Serial |  | 72 | >1  (>1)  >1 |  | 291 | 0.5  (0.25-1)  100% |  | 70 | 0.06  (0.016-0.125)  44.3% |  | 188 | 0.25  (0.125-0.5)  98.4% |  | 119 | 0.06  (0.03-0.25)  99.2% |  | 43 | 0.06  (0.03-0.125)  95.3% |  | 54 | >4  (>4)  NA |
| EUCAST |  |  | NA |  |  | (0.125-1) |  |  | (0.016-0.06) |  |  | (0.03-0.25) |  |  | (0.03-0.125) |  |  | (0.016-0.06) |  |  | NA |

OLO: olorofim, AMB: amphotericin B, ISA: isavuconazole, VRC: voriconazole, ITR: itraconazole, PSC: posaconazole and TRB. ISO: ISO standard 20776-1 (ISO).

^1^Not applicable. ^2^Some MICs are off scale at ≤0.008 mg/L.

**Supplementary table 3a**. Full list of olorofim MICs (mg/L) for the 3,075 *Aspergillus* isolates by complex in alphabetical order.

| **Species** | **Dilution method** | ***N*** | **MIC (mg/L)** | | | | | | | | | | |
| --- | --- | --- | --- | --- | --- | --- | --- | --- | --- | --- | --- | --- | --- |
|  |  |  | **0.002** | **0.004** | **0.008** | **0.016** | **0.03** | **0.06** | **0.125** | **0.25** | **0.5** | **1** | **>1** |
| *Aspergillus* section *Aspergillus* |  |  |  |  |  |  |  |  |  |  |  |  |  |
| *A. chevalieri* | ISO | 1 |  |  |  |  |  |  |  |  |  |  | 1 |
|  | Serial | 1 |  |  |  |  |  |  |  |  |  |  | 1 |
| *A. intermedius* | ISO | 1 |  |  |  |  |  |  |  |  |  |  | 1 |
|  | Serial | 2 |  |  |  |  |  |  |  |  |  |  | 2 |
| *A. montevidensis* | ISO | 2 |  |  |  |  |  |  |  |  |  | 1 | 1 |
|  | Serial | 1 |  |  |  |  |  |  |  |  |  |  | 1 |
| *A. pseudoglaucus* | ISO | 1 |  |  |  |  |  |  |  |  |  |  | 1 |
|  | Serial | 2 |  |  |  |  |  |  |  |  |  |  | 2 |
|  |  |  |  |  |  |  |  |  |  |  |  |  |  |
| ***A. candidus* complex** |  |  |  |  |  |  |  |  |  |  |  |  |  |
| *A. tritici* | Serial | 1 |  |  |  |  | 1 |  |  |  |  |  |  |
|  |  |  |  |  |  |  |  |  |  |  |  |  |  |
| ***A. circumdati* complex** |  |  |  |  |  |  |  |  |  |  |  |  |  |
| *A. circumdati* complex | ISO | 5 |  |  | 1 |  | 4 |  |  |  |  |  |  |
|  | Serial | 1 |  |  |  |  |  | 1 |  |  |  |  |  |
| *A. ochraceus* | Serial | 3 |  |  |  |  |  | 1 | 2 |  |  |  |  |
| *A. westerdijkiae* | ISO | 1 |  |  |  |  |  | 1 |  |  |  |  |  |
|  | Serial | 5 |  |  |  |  | 1 | 4 |  |  |  |  |  |
|  |  |  |  |  |  |  |  |  |  |  |  |  |  |
| ***A. cremei* complex** |  |  |  |  |  |  |  |  |  |  |  |  |  |
| *A. europaeus* | Serial | 1 |  |  |  |  | 1 |  |  |  |  |  |  |
|  |  |  |  |  |  |  |  |  |  |  |  |  |  |
| ***A. flavus* SC** |  |  |  |  |  |  |  |  |  |  |  |  |  |
| *A. flavus* | Serial | 6 |  |  |  |  | 2 | 4 |  |  |  |  |  |
| *A. flavus* complex | ISO | 22 |  |  | 4 | 13 | 4 | 1 |  |  |  |  |  |
|  | Serial | 66 |  |  |  | 3 | 38 | 25 |  |  |  |  |  |
| *A. tamarii* | Serial | 3 |  |  |  | 1 | 2 |  |  |  |  |  |  |
|  |  |  |  |  |  |  |  |  |  |  |  |  |  |
| ***A. fumigatus* complex** |  |  |  |  |  |  |  |  |  |  |  |  |  |
| *A. fumigatus* | ISO | 455 |  |  | 7 | 137 | 255 | 53 | 3 |  |  |  |  |
|  | Serial | 2047 |  |  |  | 15 | 450 | 1320 | 254 | 7 | 1 |  |  |
| *A. hiratsukae* | Serial | 3 |  |  |  |  | 3 |  |  |  |  |  |  |
| *A. lentulus* | ISO | 1 |  |  |  |  |  | 1 |  |  |  |  |  |
|  | Serial | 2 |  |  |  |  |  | 1 |  | 1 |  |  |  |
| *A. nishimurae* | Serial | 1 |  |  |  |  | 1 |  |  |  |  |  |  |
| *A. quadricinctus* | Serial | 1 |  |  |  | 1 |  |  |  |  |  |  |  |
| *A. thermomutatus* | ISO | 1 |  |  |  | 1 |  |  |  |  |  |  |  |
|  | Serial | 5 |  |  |  |  | 1 | 4 |  |  |  |  |  |
| *Neosartorya glabra* | Serial | 1 |  |  |  | 1 |  |  |  |  |  |  |  |
| *Neosartorya pseudofischeri* | Serial | 1 |  |  |  |  | 1 |  |  |  |  |  |  |

ISO: ISO standard 20776-1 (ISO).

**Supplementary table 3b**. Full list of olorofim MICs (mg/L) for the 3,075 *Aspergillus* isolates by complex in alphabetical order continued.

| **Species** | **Dilution method** | ***N*** | **MIC (mg/L)** | | | | | | | | | | |
| --- | --- | --- | --- | --- | --- | --- | --- | --- | --- | --- | --- | --- | --- |
|  |  |  | **0.002** | **0.004** | **0.008** | **0.016** | **0.03** | **0.06** | **0.125** | **0.25** | **0.5** | **1** | **>1** |
| ***A. nidulans* complex** |  |  |  |  |  |  |  |  |  |  |  |  |  |
| *A. nidulans* | Serial | 2 |  |  |  |  |  |  | 2 |  |  |  |  |
| *A. nidulans* complex | ISO | 8 |  |  |  | 3 | 3 | 2 |  |  |  |  |  |
|  | Serial | 21 |  |  |  |  |  | 16 | 5 |  |  |  |  |
| *A. quadrilineatus* | Serial | 5 |  |  |  |  |  | 2 | 3 |  |  |  |  |
| *A. spinulosporus* | ISO | 1 |  |  |  |  | 1 |  |  |  |  |  |  |
|  | Serial | 4 |  |  |  |  |  | 3 | 1 |  |  |  |  |
|  |  |  |  |  |  |  |  |  |  |  |  |  |  |
| ***A. niger* complex** |  |  |  |  |  |  |  |  |  |  |  |  |  |
| *A. aculeatinus* | ISO | 2 |  |  |  | 1 | 1 |  |  |  |  |  |  |
| *A. neoniger* | ISO | 1 |  | 1 |  |  |  |  |  |  |  |  |  |
| *A. niger* | Serial | 1 |  |  |  |  |  |  | 1 |  |  |  |  |
| *A. niger complex* | ISO | 22 |  |  |  | 2 | 11 | 8 | 1 |  |  |  |  |
|  | Serial | 111 |  |  |  |  | 2 | 37 | 58 | 13 | 1 |  |  |
| *A. tubingensis* | ISO | 35 |  |  |  | 2 | 20 | 10 | 3 |  |  |  |  |
|  | Serial | 87 |  |  |  | 1 |  | 21 | 58 | 7 |  |  |  |
| *A. welwitschiae* | ISO | 2 |  |  |  |  | 1 |  | 1 |  |  |  |  |
|  | Serial | 19 |  |  |  |  | 2 | 11 | 6 |  |  |  |  |
|  |  |  |  |  |  |  |  |  |  |  |  |  |  |
| ***A. terreus* complex** |  |  |  |  |  |  |  |  |  |  |  |  |  |
| *A. hortai* | Serial | 1 |  |  |  |  | 1 |  |  |  |  |  |  |
| *A. terreus* | ISO | 12 |  |  | 7 | 5 |  |  |  |  |  |  |  |
|  | Serial | 39 |  | 1 |  | 11 | 22 | 5 |  |  |  |  |  |
| *A. terreus* complex | ISO | 1 |  |  |  | 1 |  |  |  |  |  |  |  |
|  | Serial | 25 |  |  |  | 3 | 8 | 10 | 4 |  |  |  |  |
|  |  |  |  |  |  |  |  |  |  |  |  |  |  |
| ***A. ustus* complex** |  |  |  |  |  |  |  |  |  |  |  |  |  |
| *A. calidoustus* | ISO | 1 |  |  |  |  |  |  | 1 |  |  |  |  |
|  | Serial | 8 |  |  |  |  |  |  |  | 3 | 3 | 1 | 1 |
|  |  |  |  |  |  |  |  |  |  |  |  |  |  |
| ***A. versicolor* complex** |  |  |  |  |  |  |  |  |  |  |  |  |  |
| *A. amoenus* | ISO | 1 |  | 1 |  |  |  |  |  |  |  |  |  |
| *A. creber* | ISO | 1 |  |  | 1 |  |  |  |  |  |  |  |  |
|  | Serial | 1 |  |  |  |  | 1 |  |  |  |  |  |  |
| *A. sydowii* | ISO | 4 | 1 | 3 |  |  |  |  |  |  |  |  |  |
|  | Serial | 11 |  |  | 3 | 5 | 3 |  |  |  |  |  |  |
| *A. versicolor* | ISO | 2 |  |  | 2 |  |  |  |  |  |  |  |  |
| *A. versicolor* complex | ISO | 3 |  | 1 | 1 | 1 |  |  |  |  |  |  |  |
|  | Serial | 1 |  |  |  | 1 |  |  |  |  |  |  |  |

ISO: ISO standard 20776-1 (ISO).

**Supplementary table 4.** Full list of olorofim MICs (mg/L) for the 33 *Fusarium* isolates by increasing MIC and complex, other *Trichophyton* and rare moulds.

| **Species** | **Dilution method** | ***N*** | **MIC (mg/L)** | | | | | | | | | | | |
| --- | --- | --- | --- | --- | --- | --- | --- | --- | --- | --- | --- | --- | --- | --- |
|  |  |  | **≤0.001** | **0.002** | **0.004** | **0.008** | **0.016** | **0.03** | **0.06** | **0.125** | **0.25** | **0.5** | **1** | **>1** |
| **Other *Trichophyton*** |  |  |  |  |  |  |  |  |  |  |  |  |  |  |
| *T. benhamiae* | ISO | 1 |  |  |  | 1 |  |  |  |  |  |  |  |  |
|  | Serial | 4 |  |  |  |  | 1 | 3 |  |  |  |  |  |  |
| *T. mentagrophytes* | Serial | 4 |  |  |  | 1 | 1 | 2 |  |  |  |  |  |  |
| *T. soudanense* | ISO | 1 |  |  | 1 |  |  |  |  |  |  |  |  |  |
| *T. tonsurans* | ISO | 3 |  |  | 3 |  |  |  |  |  |  |  |  |  |
|  | Serial | 2 |  |  |  |  |  | 1 | 1 |  |  |  |  |  |
|  |  |  |  |  |  |  |  |  |  |  |  |  |  |  |
| ***Fusarium*** |  |  |  |  |  |  |  |  |  |  |  |  |  |  |
| ***F. fujikuroi* SC** |  |  |  |  |  |  |  |  |  |  |  |  |  |  |
| *F. fujikuroi* complex | ISO | 3 |  |  |  |  | 2 |  | 1 |  |  |  |  |  |
|  | Serial | 2 |  |  |  |  |  |  | 1 |  | 1 |  |  |  |
| *F. musae* | Serial | 3 |  |  |  |  |  |  |  | 1 |  | 2 |  |  |
| *F. proliferatum* | Serial | 1 |  |  |  |  |  | 1 |  |  |  |  |  |  |
| ***Fusarium oxysporum* SC** |  |  |  |  |  |  |  |  |  |  |  |  |  |  |
| *F. oxysporum ss* | Serial | 1 |  |  |  |  |  |  |  |  | 1 |  |  |  |
| *F. oxysporum* complex | ISO | 1 |  |  |  |  |  |  |  |  |  | 1 |  |  |
|  | Serial | 2 |  |  |  |  |  |  |  |  |  | 2 |  |  |
| ***F. tricinctum* SC** |  |  |  |  |  |  |  |  |  |  |  |  |  |  |
| *F. avenaceum* | Serial | 1 |  |  |  |  |  |  |  |  |  |  | 1 |  |
| ***F. dimerum* SC** |  |  |  |  |  |  |  |  |  |  |  |  |  |  |
| *F. dimerum* | ISO | 2 |  |  |  |  |  |  |  |  |  |  | 1 | 1 |
|  | Serial | 7 |  |  |  |  |  |  |  |  |  |  |  | 7 |
| ***F. solani* SC** |  |  |  |  |  |  |  |  |  |  |  |  |  |  |
| *F. petroliphilum* | ISO | 1 |  |  |  |  |  |  |  |  |  |  |  | 1 |
| *F. solani* | ISO | 1 |  |  |  |  |  |  |  |  |  |  |  | 1 |
|  | Serial | 2 |  |  |  |  |  |  |  |  |  |  | 1 | 1 |
| *F. solani* complex | ISO | 3 |  |  |  |  |  |  |  |  |  |  |  | 3 |
|  | Serial | 2 |  |  |  |  |  |  |  |  |  |  |  | 2 |
| ***F. sambucinum* SC** |  |  |  |  |  |  |  |  |  |  |  |  |  |  |
| *F. sambucinum* complex | ISO | 1 |  |  |  |  |  |  |  |  |  |  |  | 1 |
|  |  |  |  |  |  |  |  |  |  |  |  |  |  |  |
| **Rare moulds** |  |  |  |  |  |  |  |  |  |  |  |  |  |  |
| *Scedosporium apiospermum* | ISO | 2 |  |  |  | 1 |  | 1 |  |  |  |  |  |  |
|  | Serial | 12 |  |  |  |  |  | 5 | 3 | 4 |  |  |  |  |
| *Scedosporium boydii* | ISO | 2 |  |  |  | 1 | 1 |  |  |  |  |  |  |  |
|  | Serial | 1 |  |  |  |  |  |  | 1 |  |  |  |  |  |
| *Scedosporium dehoogii* | Serial | 1 |  |  |  |  | 1 |  |  |  |  |  |  |  |
| *Scedosporium* spp. | Serial | 1 |  |  |  |  |  |  |  | 1 |  |  |  |  |
| *Rasamsonia piperina* | ISO | 1 | 1 |  |  |  |  |  |  |  |  |  |  |  |
| *Microascus cirrosus* | Serial | 1 |  |  |  |  |  |  |  |  | 1 |  |  |  |

ISO: ISO standard 20776-1 (ISO).
